# Seroepidemiological investigation of Getah virus in the China-Myanmar border area from 2022-2023

**DOI:** 10.3389/fmicb.2023.1309650

**Published:** 2023-12-14

**Authors:** Hao Liu, Jin Hu, Li-Xia Li, Zi-Shuo Lu, Xiu-Tao Sun, Hui-Jun Lu, Ning-Yi Jin, Lei Zhang, Li-Na Zhang

**Affiliations:** ^1^School of Life Sciences and Engineering, Foshan University, Foshan, China; ^2^Honghe Animal Disease Prevention and Control Center, Mengzi, China; ^3^Institute of Military Veterinary Medicine, Academy of Military Medical Sciences, Changchun, China; ^4^Institute of Special Economic Animal and Plant Sciences, Chinese Academy of Agricultural Sciences, Changchun, China; ^5^Eco-Engineering Department, Guangdong Eco-Engineering Polytechnic, Guangzhou, China

**Keywords:** Getah virus, virus Isolation, sequence determination and analysis, phylogenetic analysis, seroepidemiological investigation, cattle

## Abstract

Getah Virus (GETV) is an RNA virus that is transmitted by mosquitoes and can cause disease or death in a variety of vertebrates. Its prevalence is increasingly severe in Asia. This study conducted a GETV epidemiological investigation on 1,300 bovine sera collected in the Honghe Prefecture of Yunnan Province on the China-Myanmar border from 2022 to 2023. The positive rate of GETV antibodies in bovine serum in Honghe Prefecture was determined to be 20.25% through indirect Enzyme-linked immunosorbent test (ELISA) methods. Using Real-time PCR methods to detect GETV RNA in bovine serum, the positive rate was 0.23% (3/1300), and viral nucleic acids were only detected in three bovine sera in Jianshui area in 2022. The YN2305 strain was successfully isolated from mouse neuroblastoma (N2a) cells and the complete gene sequence was obtained. All the above results indicate the existence of GETV infection in cattle in Honghe Prefecture, Yunnan Province. Homology and genetic evolution analysis found that the isolated strain has a high homology with the JL1808 strain isolated from cattle in 2018, with a nucleotide identity of 100%, and a nucleotide identity of 99.8% with the SD17-09 strain isolated from foxes in 2017. Compared with the nucleotides of 44 virus strains published in Genbank, YN2305 has multiple nucleotide site mutations in the structural gene E2 and non-structural gene NS. The nucleotide and amino acid identity of the E2 gene are 94.2−100% and 96.4−100%, respectively. Genetic evolution analysis found that this virus strain is most closely related to the bovine origin JL1808, and it is in gene group III with HuN1, Kochi-01, SD17-09, MI-110-C1, and MI-110-C2 strains that causes significant clinical symptoms in Chinese pig, fox and horse populations, belonging to the same evolutionary branch. This study determined the infection rate, genotype, and main prevalence areas of GETV in bovine sera in the China-Myanmar border area. Therefore, the epidemiological investigation of GETV infection in multiple animal hosts should be further expanded, and research on its pathogenicity and vectors should be carried out.

## 1 Introduction

Getah virus (GETV) is an arbovirus, belonging to the *Alphavirus* genus of the *Togaviridae* family, along with Chikungunya virus (CHIKV) and Venezuelan equine encephalitis virus (VEEV) (Zhai et al., 2008; Shi et al., 2022a). GETV was first isolated from *Culex gelidus* mosquitoes in Malaysia in 1955 (Zhai et al., 2008; Ren et al., 2020; Xing et al., 2020; Shi et al., 2022a; Sun et al., 2022). Since then, GETV has rapidly expanded its host range and geographical distribution (Zhai et al., 2008). Currently, it has appeared and become prevalent in 12 countries including China, Russia, South Korea, Japan, Thailand, and Australia (Zhai et al., 2008; Yang et al., 2018; Rattanatumhi et al., 2022). Since its first isolation from mosquitoes in Hainan Province, China in 1964, it has broken out and become prevalent in 17 provinces and cities (Zhai et al., 2008). So far, the virus RNA or antibodies have been detected in horses, pigs, cattle, sheep, blue foxes, kangaroos, monkeys, red pandas, and some poultry (Nemoto et al., 2015; Li et al., 2017, 2019; Yang et al., 2018; Ren et al., 2020; Xing et al., 2020; Rattanatumhi et al., 2022; Shi et al., 2022a; Zhao et al., 2022). Of particular note is that neutralizing antibodies against GETV have been found in human serum in Malaysia, Australia, and China (Zhai et al., 2008; Shi et al., 2022b), and the specific antibody titers in febrile populations are significantly higher than in healthy populations, indicating that GETV infection is related to human diseases. Yunnan Province, located in southwestern China, borders Myanmar, Laos, and Vietnam. Frequent tourism and animal trade have accelerated the spread of arboviruses locally, which may pose a threat to livestock and human health. In recent years, most GETV strains in China have come from four genera and seven species of mosquito specimens in Yunnan Province (Li et al., 2019). Cattle are the primary reservoir hosts of arboviruses, however, there have been no reports on cattle infection with GETV in the border areas of Yunnan Province. Therefore, this study conducted a GETV epidemiological investigation on bovine serum samples in the China-Myanmar border area from 2022-2023 using indirect ELISA and real-time PCR methods, to provide data support for disease prevention and control.

## 2 Materials and methods

### 2.1 Animal serum collection

From 2022 to 2023, a total of 1,300 bovine sera were collected in Honghe Prefecture, Yunnan Province, including Luxi (400), Jianshui (420), and Mile (480). All samples were stored in a −80°C freezer.

### 2.2 RNA detection

RNA was extracted from the bovine serum samples using the QIAamp Viral RNA Mini Kit (Qiagen). The RNA was then converted to cDNA using the Vazyme HiScript II First Strand cDNA Synthesis Kit (Vazyme Biotech Co., Ltd, China). The detection was carried out using the previously established quantitative reverse transcription PCR (RT-qPCR) detection method for GETV non-structural protein 1 (nsP1) (Shi et al., 2018).

### 2.3 Enzyme-linked immunosorbent test (ELISA)

The amino acid sequence of the E2 protein of GETV virus strain in Genbank was used, and after codon optimization, the gene was artificially synthesized and directionally cloned into the pGEX-6P-1 vector. After correct identification by enzyme cleavage and sequencing, it was transformed into the host bacterium BL-21 for induced expression. The identification of the recombinant E2 (rE2) protein was achieved by employing Western blot and SDS-PAG techniques subsequent to the completion of GST tag purification. Subsequently, the rE2 protein was employed as a diagnostic antigen in order to establish an indirect ELISA method for the detection of GETV antibodies, in accordance with prior literature (Sun et al., 2022). The cross-reactivity of the rE2-based indirect ELISA was assessed by examining the anti-sera from various common bovine and porcine viruses, such as BVDV, FMDV, PRRSV, BATV, JEV, and PEDV. Additionally, the sensitivity of the Indirect ELISA was analyzed by testing different dilutions of bovine GETV positive serum.

### 2.4 Virus isolation and amplification of whole gene sequence

The serum with GETV nucleic acid positive was filtered through a 0.22°μm filter (Millipore, Billerica, MA, USA) and inoculated into N2a monolayer cells in a six-well plate. After incubating at 37°C for 1°h, the cells were washed twice with PBS, and preserved in 2% FBS (Gibco) MEM in a 5% CO_2_ incubator. The cytopathic effect (CPE) of the cells was observed daily. After five continuous cell passages, the cell suspension was collected and the isolated GETV strain was sequenced for the whole genome (Ren et al., 2020).

### 2.5 Alignment and phylogenetic analysis of GETV genome sequences

To analyze the homology relationship between GETV YN2305 isolate and other various GETV strains, the MegAlign software was used to perform homology analysis on the full gene and E2 gene sequences of the YN2305 isolate. Other GETV strains are from those registered in Genbank. MEGA 7 software was used to perform phylogenetic analysis on 44 full genes and 58 E2 gene groups of GETV that have been published in Genbank and YN2305. The Tamura-Nei model and the maximum likelihood method of gamma distribution rate heterogeneity were used in MEGA 7.

### 2.6 Statistical analysis

The binom.confint function in R version 3.5.0 was used to calculate the seroprevalence and 95% confidence interval (95% CI).

## 3 Results

### 3.1 Results of the serological tests

From 2022 to 2023, a total of 1,300 bovine sera were collected in Honghe Prefecture, Yunnan Province, with a total positive rate of 20.25% (108/534, 95% CI: 17.04−27.84). Among them, 1,000 bovine serum samples collected in Jianshui, Mile, and Luxi regions of Honghe Prefecture in Yunnan Province in 2022 were tested, with a positive rate of 20.78% (53/255, 95% CI: 16.26−26.18). The positive rates in each region were 24.74% (23/93, 95% CI: 17.08−34.38), 12.17% (9/74, 95% CI: 6.53−21.53), and 23.86% (21/88, 95% CI: 16.17−33.74), respectively. In 2023, the positive rate of GETV antibodies in 300 bovine serum samples collected in Honghe Prefecture was 19.71% (55/279, 95% CI: 15.47−24.78), with Jianshui, Mile, and Luxi counties at 8.6% (8/93, 95% CI: 4.42−16.07), 36.56% (34/93, 95% CI:27.49−46.7), and 13.98% (13/93, 95% CI: 8.35−22.46), respectively, (Table 1).

**TABLE 1:** The positive rate of GETV in bovine serum in 2022-2023.

Species	Region	Year			
		2022		2023	
		Positive rate	95% CI	Positive rate	95% CI
Cattle	JS	24.74 (23/93)	17.08−34.38	8.6 (8/93)	4.42−16.07
	ML	12.17 (9/74)	6.53−21.53	36.56 (34/93)	27.49−46.7
	LX	23.86 (21/88)	16.17−33.74	13.98 (13/93)	8.35−22.46
	Total	20.78 (53/255)	16.26−26.18	19.71 (55/279)	15.47−24.78

*JS, Jianshui County; ML, Mile City; LX, Luxi County.

### 3.2 GETV RNA test results

All bovine serum samples were tested for GETV RNA using RT-qPCR method, with a positive rate of 0.23% (3/1300).

### 3.3 Enzyme-linked immunosorbent test (ELISA)

Following the process of GST label purification, the E2 protein underwent identification through SDS-PAGE and Western blot techniques, which revealed a protein size of 74.6 kDa, aligning with the anticipated value (Technical Appendix Supplementary Figure 1). The average outcomes of the antisera for BVDV, FMDV, PRRSV, BATV, JEV, and PEDV indicated negativity, thereby confirming the absence of cross-reactivity with the established rE2 indirect ELISA. Consequently, the indirect ELISA exhibited a significant of specificity. In addition, the serum from bovines infected with GETV were subjected to testing at various dilutions, ranging from 1:100 to 1:51200. The results indicated that the lowest detectable dilution using this methodology was 1:6400.

### 3.4 GETV homology and genetic evolution analysis

**FIGURE 1:**
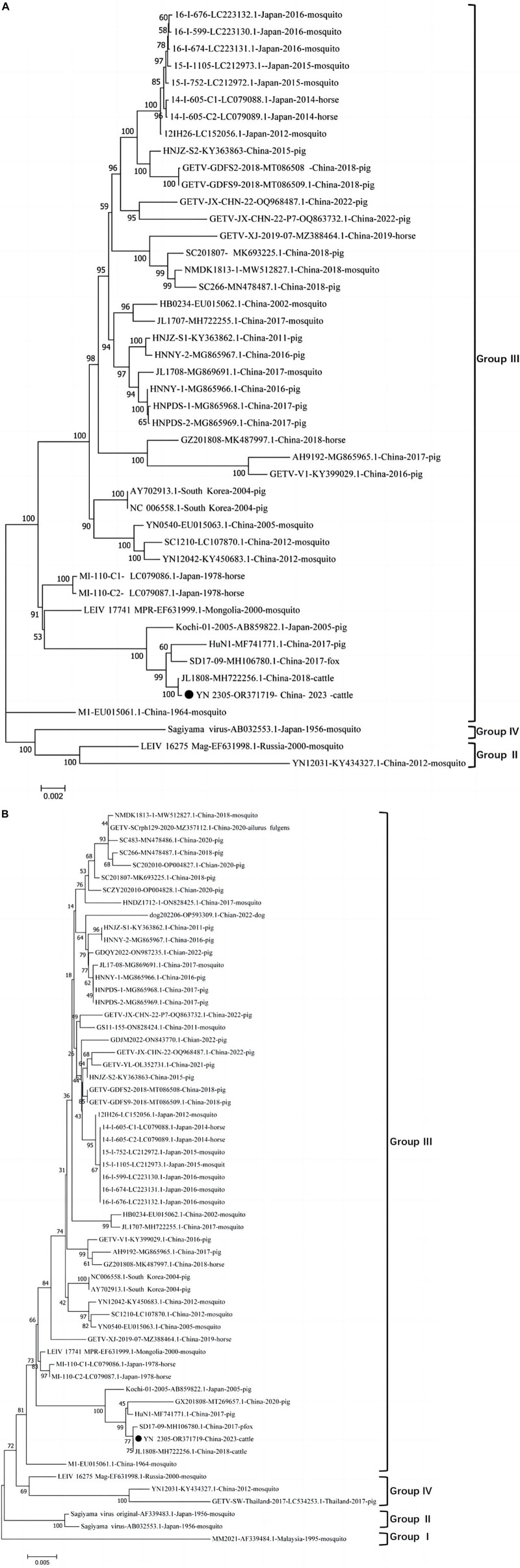
**(A)** Diagrammatic representation of a tree based on the complete genome sequence of GETV YN2305 and the available complete GETV genomes from the GenBank. **(B)** Diagrammatic representation of a tree based on the complete E2 gene nucleotide sequences of GETVs.

The full gene sequence of GETV YN2305 strain was obtained by PCR method with a length of 11,689 nt and submitted to Genbank (Genbank number: OR371719). Homology analysis was carried out with 44 GETV strains registered in Genbank, revealing that the YN2305 strain has the highest nucleotide homology with the bovine-derived JL1808 strain, with an identity of 100%. The nucleotide identity with the fox-derived SD17-09 strain and pig-derived Kochi-01 strain were 99.8 and 99.5%, respectively. Similarly, comparison with 58 E2 gene nucleotide sequences revealed that the identity of YN2305 strain with JL1808 was 100%, while the identity with E2 genes of other strains was 94.2−99.8% (Table 2). Through the phylogenetic analysis of the full gene and E2, YN2305 belongs to the GETV gene group III, and is most closely related to the bovine-derived JL1808 in genetic evolution, belonging to the same branch (Li et al., 2017; Figures 1A, B).

**TABLE 2:** Nucleotide sequences and identity analysis of YN2305 and the other GETV strains.

Virus isolates	YH2305 (%)	
	E2 genome	Complete genome
	nt	nt
AY702913.1	97.6	98.1
GETV-GDFS2	97.4	97.6
GETV-GDFS9	97.4	97.6
GETV-JX-CHN-22	96.9	97.5
GETV-JX-CHN-22-P7	97.2	97.5
GETV-V1	97.3	97.8
GETV-XJ	97.4	97.7
GZ201808	97.2	97.6
HB0234	96.8	97.9
HNJZ-S1	97.2	97.8
HNJZ-S2	97.4	97.8
HNNY-1	97.3	97.8
HNNY-2	97.2	97.8
HNPDS-1	97.3	97.8
HNPDS-2	97.3	97.8
HuN1	99.7	99.5
JL1707	97.2	97.8
JL1708	96.8	97.8
JL1808	100	100
Kochi-01	99.2	99.5
LEIV 16275 Mag	97.2	97.5
LEIV 17741 MPR	98.2	98.6
M1	97.4	98
MI-110-C1	98	98.5
MI-110-C2	98.1	98.5
NC_006558.1	97.6	98.1
NMDK1813-1	97.2	97.6
Sagiyama virus	96.4	97.2
SC266	97.2	97.5
SC1210	97.1	97.8
SC201807	97.5	97.7
SD17-09	99.8	99.8
YN0540	97.3	97.9
YN12031	95.8	96.3
YN12042	97.2	97.8
12IH26	97.4	97.8
14-I-605-C1	97.3	97.7
14-I-605-C2	97.3	97.7
15-I-752	97.3	97.7
15-I-1105	97.3	97.7
16-I-599	97.3	97.7
16-I-674	97.3	97.7
16-I-676	97.3	97.7
AH9192	97.1	97.6
Sagiyama virus original	96.6	—
dog202206	96.5	—
GDJM2022	97.2	—
GDQY2022	97.3	—
GETV-SCrph129	97.3	—
GETV-SW	95.1	—
GETV-YL	97.2	—
GS11-155	97.2	—
GX201808	99	—
HNDZ1712-1	97.2	—
MM 2021	94.2	—
SC202010	96.9	—
SC483	97.3	—
SCZY202010	97.3	—

## 4 Discussion

Getah virus (GETV) was first discovered in Hainan Province, China in 1964 (Li et al., 2019; Lu et al., 2019). In recent years, the widespread distribution of GETV has been found in mosquitoes and animals in 21 provinces and cities including Yunnan, Sichuan, Guizhou, Gansu, Jilin, Hebei, and Shanxi. Prior to 2006, only six provinces in China were known to be affected by GETV (Zhai et al., 2008), but by 2018, the number of provinces affected had dramatically increased to 15 (Lu et al., 2019; Xia et al., 2021). Yunnan Province, bordering Laos, Myanmar, and Vietnam, has frequent trade and a climate conducive to mosquito breeding, facilitating the transmission of GETV. Reports indicate that there are high titers of GETV neutralizing antibodies in many livestock in Yunnan Province, and the positive antibody rate in pigs is high (Li et al., 2019), indicating that cattle, as livestock, face the risk of infection from pig-derived GETV. This study detected the highest positive rate of GETV antibodies in bovine serum in Jianshui County, Honghe Prefecture, Yunnan Province in 2022 at 24.74% using indirect ELISA methods. In 2023, the positive rate in Jianshui decreased by 16.14%, which related to the implementation of local pest and mosquito control measures and the seasonal prevalence of vector-borne viruses. While the positive rate in Mile County increased by 24.39% compared to the previous year. Based on these serological survey results, we speculate that there is a trend for GETV virus to spread from border areas to inland areas. The YN2305 strain was successfully isolated from cattle in Yunnan Province (Genbank Number: OR371719), and this isolate has the highest nucleotide identity and the closest genetic evolutionary relationship with the previously isolated bovine-derived JL1808 strain in Jilin Province. It also has high identity and close phylogenetic relationships with the fox-derived SD17-09 strain and the pig-derived HuN1 strain, which causes fever, anorexia, and neurological symptoms in host animals, but there are multiple mutations in the E2 gene and NS gene of GETV, which may be related to host differences (Shi et al., 2022b). In addition, phylogenetic analysis shows that YN2305, along with Kochi-01, MI-110-C1, and MI-110-C2 strains that causes significant clinical symptoms in Chinese pig and horse populations, belongs to gene group III and the same evolutionary branch (Nemoto et al., 2015). These results indicate that GETV is widely present in various animal hosts with differences in nucleotide sequences. As cattle are in close contact with humans and other animals, the bovine-derived isolate YN2305 may pose a threat to other livestock, wildlife, and even humans. To reduce the impact of GETV virus on humans and animals, it is necessary to further expand the scope of monitoring and carry out long-term serological surveys.

## 5 Conclusion

In conclusion, this study indicates that GETV is present and prevalent in cattle in the China-Vietnam border region, with the highest positive rate of bovine serum antibodies in Jianshui and Mile counties, and a trend to spread inland. In addition, GETV YN2305 strain has the highest homology with bovine isolate JL1808, but there are genetic differences in the E2 and NS genes with closely related fox and pig derived isolates. The isolate may pose a threat to other livestock and humans. This study fills the gap in information on the infection rate and prevalence of GETV in livestock in the Yunnan border region, providing data support for the prevention and control of the epidemic.

## Data availability statement

The original contributions presented in the study are publicly available. This data can be found here: https://www.ncbi.nlm.nih.gov/; OR371719.

## Ethics statement

The animal studies were approved by the Institutional Animal Care and Use Ethics Committee (IACUC) of the Chinese Academy of Military Medical Science. The studies were conducted in accordance with the local legislation and institutional requirements. Written informed consent was obtained from the owners for the participation of their animals in this study.

## Author contributions

HL: Funding acquisition, Investigation, Writing – original draft. JH: Investigation, Writing – original draft, Data curation, Formal Analysis. L-XL: Software, Validation, Writing – review & editing. Z-SL: Software, Validation, Writing – review & editing. X-TS: Resources, Writing – review & editing. H-JL: Resources, Writing – review & editing. N-YJ: Resources, Supervision, Writing – review & editing. LZ: Supervision, Writing – review & editing. L-NZ: Supervision, Writing – review & editing, Funding acquisition.
