# Supplementary material for: Seroepidemiological investigation of Getah virus in the China-Myanmar border area from 2022-2023
Source: Front Microbiol. 2023 Dec 14;14:1309650. doi: 10.3389/fmicb.2023.1309650 (PMC10755881; doi:10.3389/fmicb.2023.1309650)
Supplement: Supplementary file 1 [file Data_Sheet_1.docx]

Supplementary Material

Seroepidemiological Investigation of Getah Virus in the China-Myanmar Border Area from 2022-2023

Hao Liu^1†^, Jin Hu^1†^, Li-Xia Li^1^, Zi-Shuo Lu^1^, Xiu-Tao Sun^2^, Hui-Jun Lu^3^, Ning-Yi Jin^3^, Lei Zhang^4*^ , Li-Na Zhang^5*^

***Correspondence:** Li-Na Zhang
zln_tiantang@163.com

Lei Zhang
zhanglei_tcs@126.com

# Supplementary Figures

A

B


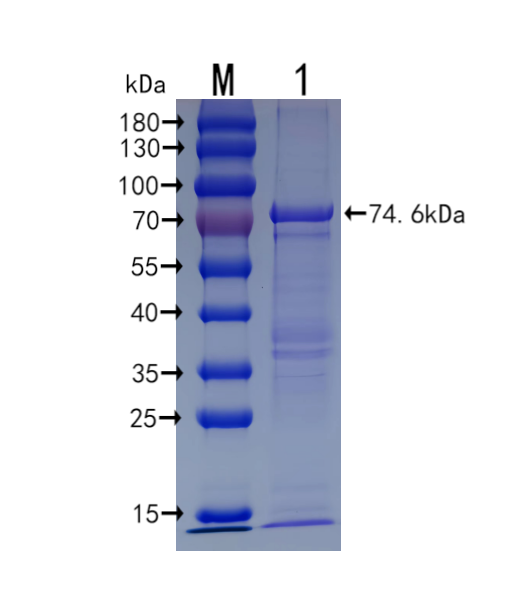

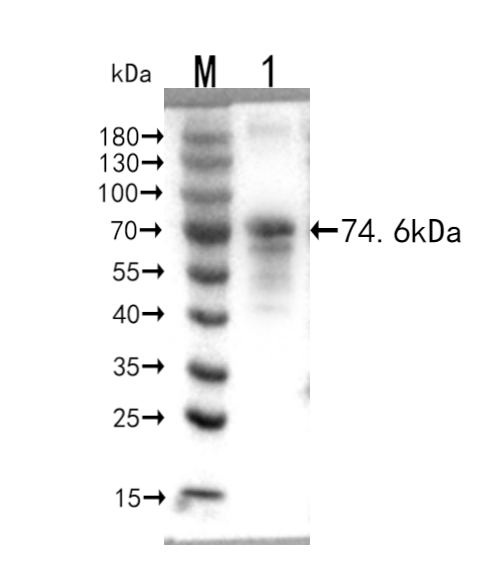


**Figure S1.** (A) SDS-PAGE analysis of E2 protein expressed in Escherichia coli. The E2 protein was 74.6kDa on SDS-PAGE.M,Marker;lane1:Purified target protein.(B)Western blotting analysis of E2 protein with an anti-His antibody.M,Marker;lane 1:Purified target protein.
